# HOW IS THE NURSING HOME QUALITY OF CARE ALIGNED WITH THE QUALITY OF LIFE?

**DOI:** 10.1093/geroni/igad104.3398

**Published:** 2023-12-21

**Authors:** Jenny Kwon, John Bowblis

**Affiliations:** Miami University, Oxford, Ohio, United States; Miami University, Oxford, Ohio, United States

## Abstract

The CMS Five-Star Quality Rating System does not currently incorporate nursing home (NH) consumers’ opinions. This study aims to investigate the association between star ratings and resident/family satisfaction. A unique, facility-level dataset of Ohio NHs that contains star ratings, consumer satisfaction scores, and NH characteristics was constructed (N=701). The key dependent variables were the overall, health inspection, and quality star ratings, categorized into two groups: 1-3 stars and 4-5 stars. Key independent variables were overall resident and family satisfaction scores, which ranged from 0 (low) to 100 (high). Bivariate and logistic regression analyses were conducted. Bivariate results showed consumer satisfaction and star ratings were correlated, but not perfectly related. Regression results found an increase in resident satisfaction was associated with an increased probability of being a 4-5 star rated facility across all star ratings (p<.05). An increase in family satisfaction was also associated with an increased probability of being a 4-5 star rated facility for the overall and health inspection ratings (p<.01). Early studies found little relationship between star ratings and consumer satisfaction. Due to improvements made to the star ratings, this study found consumer satisfaction is associated, though imperfectly, with star ratings, suggesting that a consumer-driven measure of quality still needs to be publicly reported.

